# Prognostic role of tumor-infiltrating lymphocytes in gastric cancer: a meta-analysis

**DOI:** 10.18632/oncotarget.18065

**Published:** 2017-05-22

**Authors:** Xiao Zheng, Xing Song, Yingjie Shao, Bin Xu, Lujun Chen, Qi Zhou, Wenwei Hu, Dachuan Zhang, Changping Wu, Min Tao, Yibei Zhu, Jingting Jiang

**Affiliations:** ^1^ Department of Tumor Biological Treatment, The Third Affiliated Hospital of Soochow University, Changzhou 213003, People's Republic of China; ^2^ Jiangsu Engineering Research Center for Tumor Immunotherapy, Changzhou 213003, People's Republic of China; ^3^ Institute of Cell Therapy, Soochow University, Changzhou 213003, People's Republic of China; ^4^ Department of Radiation Oncology, The Third Affiliated Hospital of Soochow University, Changzhou 213003, People's Republic of China; ^5^ Department of Oncology, The Third Affiliated Hospital of Soochow University, Changzhou 213003, People's Republic of China; ^6^ Department of Pathology, The Third Affiliated Hospital of Soochow University, Changzhou 213003, People's Republic of China

**Keywords:** TILs, prognosis, gastric cancer, biomarker, meta-analysis

## Abstract

**Background:**

In patients with gastric cancer, the prognostic value of tumor-infiltrating lymphocytes (TILs) is still controversial. A meta-analysis was performed to evaluate the prognostic value of TILs in gastric cancer.

**Materials and methods:**

We identify studies from PubMed, Embase and the Cochrane Library to assess the prognostic effect of TILs in patients with gastric cancer. Fixed-effects models or random-effects models were used estimate the pooled hazard ratios (HRs) for overall survival (OS) and disease-free survival (DFS), which depend on the heterogeneity.

**Results:**

A total of 31 observational studies including 4,185 patients were enrolled. For TILs subsets, the amount of CD8^+^, FOXP3^+^, CD3^+^, CD57^+^, CD20^+^, CD45RO^+^, Granzyme B^+^ and T-bet^+^ lymphocytes was significantly associated with improved survival (*P* < 0.05); moreover, the amount of CD3+ TILs in intra-tumoral compartment (IT) was the most significant prognostic marker (pooled HR = 0.52; 95% CI = 0.43–0.63; *P* < 0.001). However, CD4^+^ TILs was not statistically associated with patients’ survival. FOXP3+ TILs showed bidirectional prognostic roles which had positive effect in IT (pooled HR = 1.57; 95% CI = 1.04–2.37; *P* = 0.033) and negative effect in extra-tumoral compartment (ET) (pooled HR = 0.76; 95% CI = 0.60–0.96; *P* = 0.022).

**Conclusions:**

This meta-analysis suggests that some TIL subsets could serve as prognostic biomarkers in gastric cancer. High-quality randomized controlled trials are needed to decide if these TILs could serve as targets for immunotherapy in gastric cancer.

## INTRODUCTION

Gastric cancer has become a big health issue worldwide due to high morbidity and mortality [1]. Although the incidence and mortality of gastric cancer has been decreasing over the past decade, it is still the third cause of cancer death in men and the second in women in China [2]. Besides early diagnosis, appropriate treatment plans identified from the prediction of patients’ outcomes also contribute to successful treatment of gastric cancer. Therefore, further investigation is needed to identify newer tumor biomarkers with higher specificity and sensitivity in gastric cancer to determine the optimal therapeutic strategies and predict the prognosis of gastric cancers.

Tumor-infiltrating lymphocytes (TILs) are a heterogeneous group containing tumor-infiltrating T cells, B cells, and natural killer (NK) cells [3]. There are many specific antigens such as CD3, CD4, CD8, FOXP3, CD20, CD57 in the cell membrane of TILs. Usually, different cell surface antigens bind to specific type of lymphocytes. For example, CD3, CD4, CD8, FOXP3 bind to T cells; CD20 binds to B cells; and CD57 binds to NK cells. Thus, TILs play a bidirectional regulation roles in the tumor-associated immune responses: It can not only inhibit tumor growth by suppressing their outgrowth or destroying cancer cells, but also contribute to tumor progression either by creating a tumor microenvironment that stimulate tumor outgrowth or by selectively protecting tumor cell survival in an immunocompetent host [4].

TILs might be a prognostic biomarker in gastric cancer. Many studies have verified TILs for use in prognostic prediction besides their crucial role in tumor-associated immune responses in gastric cancer [5–8]. Lee et al. found that high infiltration of CD8^+^T cells was associated with better outcome [5], whereas no correlation between the infiltration of CD8^+^ T cells and clinical outcome was found in Hass et al. study [6]. Zhou et al. revealed that patients with high density of FOXP3^+^ T cell had worse overall survival (OS) [7], but Kim et al. reported the opposite results [8]. Therefore, the prognostic role of TILs in gastric cancer remains controversial. It is also unclear whether the associations between the density of TILs and prognosis vary depending on survival-associated factors such as the endpoints and anatomical location. In view of the above-mentioned facts, to assess the prognostic effect of TILs in gastric cancer, we performed this meta-analysis.

## RESULTS

### Study characteristics

**Figure 1:**
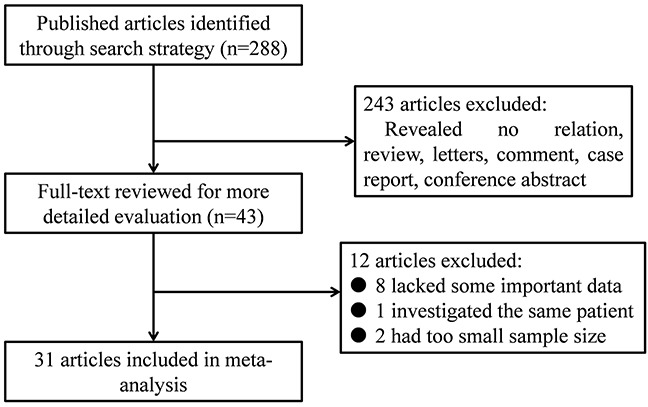
Flow diagram of the study selection process

A total of 288 references were initially collected in our study. Among them, 43 articles reported the association between the density of TILs and patients’ outcomes in gastric cancer. After further screening, 12 articles were excluded (one studying the same population, three lacking enough sample size, and eight without some useful data). Finally, we incorporated 31 articles including 4,185 patients from Italy, Japan, Germany, China, Korea, Turkey and France into this meta-analysis (Figure 1) [5–35].

The main characteristics of studies were showed in Table 1. Among these, only five articles, including 875 patients with gastric cancer, reported the prognostic value of generalized TILs [9, 10, 13, 15, 29], and others focused on specific TIL subsets. The prognostic role of TILs in intra-tumoral compartment (IT) and extra-tumoral compartment (ET) were evaluated in 31 and 5 articles, respectively. 20 articles have large sample size (≥100). The details of tumor stage were provided in 21 articles, and the categorizations were various. Follow-up time was available in 27 articles, and the number of articles with median follow-up of more than 60 months was 11. The cut-off values contained mean value (n = 5), median value (n = 17), and others. Specific TIL subsets and generalized TILs were detected by means of immunohistochemistry (IHC) staining and Hematoxylin-eosin (HE) staining, respectively. 23 studies reported the Hazard ratios (HRs) directly, but the other 8 studies only provided Kaplan-Meier Survival curves. The endpoints OS and disease-free survival (DFS) were provided in 27 and 9 studies, respectively.

**Table 1:** Main characteristics of all studies included in the meta-analysis

Study	Subset	Location	Country	Case number	Tumor stage	Follow-up (months)	Detected method	Cut-off value	HRs provided from	Outcome
Chiaravalli 2005 [11]	CD3^+^	IT	Italy	96	27/31/30/8	64.3(0-238)	IHC	median	sc	OS
	CD8^+^	IT	Italy	71	NR	64.3(0-238)	IHC	median	sc	OS
Ishigami 1999 [29]	CD57^+^	IT	Japan	146	54/26/27/39	87	IHC	>25 cells/HPF	report	OS
	TILs	IT	Japan	146	54/26/27/39	87	HE	>150 cells/HPF	report	OS
Ohno 2002 [27]	CD8^+^	IT	Japan	84	I-II31/III-IV53	38.3(2.9-109)	IHC	median	report	DFS
Gochi 2001 [9]	TILs	IT	Japan	159	NR	>120	HE	moderate or marked	report	OS
Haas 2009 [6]	CD3^+^	Primary/ Metastasis/ Normal	Germany	52	20/19/10/3	71.2(54.7-57.8)	IHC	mean	report	OS
	CD8^+^	Primary/ Metastasis/ Normal	Germany	52	20/19/10/3	71.2(54.7-57.8)	IHC	mean	report	OS
	CD20^+^	Primary/ Metastasis	Germany	52	20/19/10/3	71.2(54.7-57.8)	IHC	mean	report	OS
	FOXP3^+^	Primary/ Metastasis/ Normal	Germany	52	20/19/10/3	71.2(54.7-57.8)	IHC	mean	report	OS
	Granzyme B^+^	Primary/ Metastasis/ Normal	Germany	52	20/19/10/3	71.2(54.7-57.8)	IHC	mean	report	OS
Dong 2013 [19]	CD8^+^	IT	China	100	4/55/38/3	36.5(2-88)	IHC	median	report	OS/DFS
	CD20^+^	IT	China	100	4/55/38/3	36.5(2-88)	IHC	median	report	OS/DFS
Ishigami 2001 [28]	CD3^+^	IT	Japan	185	86/36/34/27	>60	IHC	66%	sc	OS
Kijima 2003 [34]	CD8^+^	IT	Japan	66	NR	30(1-176)	IHC	≥50 cells/HPF	report	OS
	CD57^+^	IT	Japan	64	NR	30(1-176)	IHC	≥25 cells/HPF	report	OS
Ishigami 2000 [30]	CD57^+^	IT	Japan	169	NR	63(13-122)	IHC	≥25 cells/HPF	sc	OS
Mizukami 2007 [33]	FOXP3^+^	IT	Japan	80	42/12/15/11	87.7	IHC	median	report	OS
Lee 2008 [5]	CD3^+^	IT	Korea	220	67/53/55/45	64.4(1-108)	IHC	mean	report	OS
	CD8^+^	IT	Korea	220	67/53/55/45	64.4(1-108)	IHC	mean	report	OS
	CD45RO^+^	IT	Korea	220	67/53/55/45	64.4(1-108)	IHC	mean	report	OS
Perronea 2008 [16]	FOXP3^+^	IT	Italy	110	II46/III46	>36	IHC	median	report	OS/DFS
Shen 2010 [35]	CD4^+^	IT / Peri-tumor/ TDLN	China	133	I-II66/III-IV67	43(36-104)	IHC	median	report	OS
	CD8^+^	IT / Peri-tumor/ TDLN	China	133	I-II66/III-IV67	43(36-104)	IHC	median	report	OS
	FOXP3^+^	IT / Peri-tumor/ TDLN	China	133	I-II66/III-IV67	43(36-104)	IHC	median	report	OS
Wang 2011 [14]	FOXP3^+^	IT / Peri-tumor / Normal	China	107	33/21/21/32	62(2-120)	IHC	median	report	OS
Lu 2011 [32]	FOXP3^+^	IT	China	60	NR	NR	IHC	median	sc	OS
Kim 2011 [17]	CD3^+^	IT	China	180	I-II91/III89	median 45	IHC	median	report	OS/DFS
	CD4^+^	IT	China	180	I-II91/III89	median 45	IHC	median	report	OS/DFS
	CD8^+^	IT	China	180	I-II91/III89	median 45	IHC	median	report	OS/DFS
	FOXP3^+^	IT	China	180	I-II91/III89	median 45	IHC	median	report	OS/DFS
	Granzyme B^+^	IT	China	180	I-II91/III89	median 45	IHC	median	report	OS/DFS
Ishigami 2012 [31]	FOXP3^+^	IT	Japan	141	NR	NR	IHC	mean	sc	OS
Chen 2012 [24]	T-bet^+^	IT	China	152	10/31/93/18	NR	IHC	NR	report	OS/DFS
Diricana 2013 [10]	TILs	IT	Turkey	52	NR	NR	HE	NR	sc	OS
Kim 2013 [8]	CD8^+^	IT	Korea	99	I-II60/III-IV39	59(1-96)	IHC	median	report	OS
	FOXP3^+^	IT	Korea	99	I-II60/III-IV39	59(1-96)	IHC	median	report	OS
Kang 2015 [13]	TILs	IT	Korea	120	74/26/19/1	22.2(2.1-50.8)	HE	median	report	DFS
Giampieri 2015 [26]	CD3^+^	IT	Italy	103	NR	>12	IHC	≥50-60%	report	OS
Dai 2016 [15]	TILs	IT	China	398	I-II132/III-IV262	61.2(12.2-79.9)	HE	NR	report	OS
Chen 2011 [18]	CD8^+^	IT	China	192	8/71/94/19	61(0.3-81.6)	IHC	median	report	OS
Hu 2014 [25]	FOXP3^+^	IT	China	56	I-II16/III-IV40	19(1-52)	IHC	median	Repor-t	OS
Li 2015 [20]	CD8^+^	IT	China	192	I-II48/III-IV144	19.5(1-54)	IHC	median	report	OS
	CD4^+^	IT	China	192	I-II48/III-IV144	19.5(1-54)	IHC	median	report	OS
Hennequin 2015 [12]	CD20^+^	IT	France	82	I-III	median 27	IHC	median	report	DFS
	CD8^+^	IT / Peri-tumor	France	82	I-III	median 27	IHC	median	report	DFS
	FOXP3^+^	IT / Peri-tumor	France	82	I-III	median 27	IHC	median	report	DFS
	T-bet^+^	IT / Peri-tumor	France	82	I-III	median 27	IHC	median	report	DFS
Liu 2015 [21]	CD3^+^	IT / Peri-tumor	China	166	23/41/80/22	median 65.8	IHC	median	report	OS
	CD4^+^	IT / Peri-tumor	China	166	23/41/80/22	median 65.8	IHC	median	report	OS
	CD8^+^	IT / Peri-tumor	China	166	23/41/80/22	median 65.8	IHC	median	report	OS
	CD57^+^	IT / Peri-tumor	China	166	23/41/80/23	median 65.9	IHC	median	report	OS
	FOXP3^+^	IT / Peri-tumor	China	166	23/41/80/23	median 65.9	IHC	median	report	OS
Wakatsuki 2012 [22]	CD45RO^+^	IT	Japan	74	16/25/15/18	NR	IHC	mean	sc	OS/DFS
Zhou 2013 [7]	FOXP3^+^	IT	China	133	NR	43(36-104)	IHC	mean	report	OS
Kim 2015 [23]	CD8^+^	IT	Korea	143	NR	70(2-111)	IHC	median	sc	DFS
	FOXP3^+^	IT	Korea	143	NR	70(2-111)	IHC	median	sc	DFS

TILs: tumor-infiltrating lymphocytes; IT: intra-tumoral compartment; IHC: immunohistochemistry; TDLN: tumor draining lymph node; HE: Hematoxylin-eosin; OS: overall survival; DFS: disease-free survival; HR: hazard ratio; NR: not report; SC: survival curve.

### Subgroup analysis

Different TIL subsets have different functions in the process of anti-tumor immunoreaction response. Therefore, subgroup analyses based on TIL subsets were performed. Then, given that the function of TILs is associated with the distribution site, we further performed subgroup analyses based on the distribution site of TILs. Table 2 shows the major results of subgroup analyses.

**Table 2:** The pooled associations between TILs subsets and the prognosis of patients with gastric cancer

Subset/Outcome	Location	Study number	Case number	HR (95%CI)-model	*P* value	Heterogenety	
						*I*^2^ (%)	*P*
Generalized TILs							
OS	IT	4	755	0.55 (0.35-0.86) - random	0.009	62.4	0.047
DFS	IT	1	120	0.21 (0.04-1.09)	0.063		
CD8^+^							
OS	IT	12	1523	0.73 (0.61-0.87) - fixed	<0.001	0	0.622
	ET	4	484	0.71 (0.53-0.97) - fixed	0.029	0	0.583
DFS	IT	5	589	0.57 (0.35-0.94) - random	0.029	79.5	0.001
	ET	1	84	0.98 (0.50-2.00)	0.950		
FOXP3^+^							
OS	IT	12	1369	1.57 (1.04-2.37) - random	0.033	71.6	<0.001
	ET	6	698	0.76 (0.60-0.96) - fixed	0.022	0	0.519
DFS	IT	4	516	1.09 (0.64-1.86) - random	0.756	73.8	0.010
	ET	1	85	1.23 (0.60-2.50)	0.500		
CD3^+^							
OS	IT	8	1054	0.52 (0.43-0.63) - fixed	<0.001	18.6	0.283
	ET	2	218	0.80 (0.38-1.65) - random	0.542	50.1	0.157
DFS	IT	1	180	0.70 (0.43-1.15)	0.163		
CD4^+^							
OS	IT	4	671	0.82 (0.54-1.24) - random	0.353	60.9	0.053
	ET	3	432	0.95 (0.57-1.57) - random	0.829	57.7	0.094
DFS	IT	1	180	0.71 (0.41-1.24)	0.225		
CD57^+^							
OS	IT	4	545	0.89 (0.85-0.94) - fixed	<0.001	29.1	0.237
	ET	1	166	0.73 (0.47-1.14)	0.162		
CD20^+^							
OS	IT	3	204	0.73 (0.24-2.25) - random	0.588	71.5	0.030
DFS	IT	2	182	0.54 (0.34-0.86) - fixed	0.010	0	0.364
CD45RO^+^							
OS	IT	2	294	0.56 (0.37-0.84) - fixed	0.005	0	0.526
DFS	IT	1	74	0.58 (0.29-1.18)	0.096		
Granzyme B^+^	IT						
OS	IT	3	284	0.82 (0.52-1.28) - fixed	0.376	0	0.975
	ET	1	52	0.35 (0.12-0.99)	0.050		
DFS	IT	1	180	0.99 (0.98-1.01)	0.319		
T-bet^+^	IT						
OS	IT	1	152	0.55 (0.30-0.99)	0.047		
DFS	IT	2	235	0.54 (0.34-0.87)	0.012	0	0.833
	ET	1	86	0.90 (0.45-1.80)	0.800		

OS: overall survival; DFS: disease-free survival; HR: hazard ratio; CI: confidence interval; IT: intra-tumoral compartment; ET: extra-tumoral compartment.

### Generalized TILs

**Figure 2:**
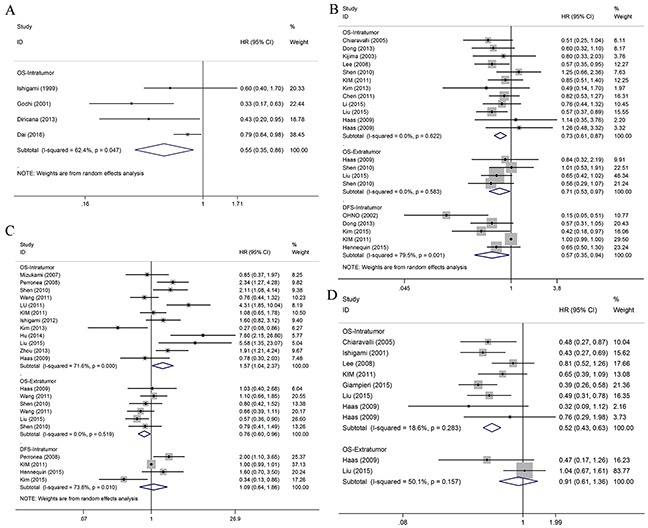
Forest plots of studies evaluating hazard ratios of high level of TILs subsets in gastric cancer by the distribution **(A)** Forest plot for the prognostic effect of general TILs. **(B)** Forest plot for the prognostic effect of CD8^+^ T cells. **(C)** Forest plot for the prognostic effect of FOXP3^+^ T cells. **(D)** Forest plot for the prognostic effect of CD3^+^ T cells.

Five articles focused on the clinical outcome of patients with high density of generalized TILs in IT [9, 10, 13, 15, 29]. Four of the articles evaluated OS [9, 10, 15, 29], and the pooled HR was 0.55 (95% CI = 0.35-0.86; *P* = 0.009) (Figure 2A). Only one of them evaluated DFS [13], and there was no statistical significance between high density of generalized TILs and DFS in gastric cancer (*P* = 0.63).

### CD8^+^ T lymphocyte subset

In total, 14 articles including 1,803 patients with gastric cancer researched the clinical outcome of patients with high density of CD8^+^ TILs [5, 6, 8, 11, 12, 17–21, 23, 27, 34, 35]. Stratified by the endpoints, there were 11 of OS and 5 of DFS, respectively.

### OS

Eleven articles focused on the OS of patients with high density of CD8^+^ TILs in IT [5, 6, 8, 11, 17–21, 34, 35]. The pooled analysis revealed that high density of infiltration of CD8^+^ T lymphocytes was statistically significant associated with better OS (pooled HR = 0.73; 95% CI = 0.61-0.87; *P* < 0.001) (Figure 2B). Subgroup analysis showed that patients with high infiltration of CD8^+^ T lymphocytes had better OS in the groups with large sample size (≥100; pooled HR = 0.73, 95% CI = 0.60- 0.88; *P* = 0.001), Asian patients (pooled HR = 0.72, 95% CI = 0.60-0.87; *P* = 0.001), and median cut-off values (pooled HR = 0.73, 95% CI = 0.60-0.89; *P* = 0.002). However, other subgroups showed no statistical significance (Table 3). We further performed a sensitivity analysis which demonstrated the result pattern was not impacted by any single study. The results of meta-regression showed that ethnicity (*P* = 0.838), sample size (*P* = 0.996) and cut-off value (*P* = 0.959) all had no statistically significant influence on the combined effect size for OS.

**Table 3:** Pooled hazard ratios for OS according to subgroup analyses

OS-Intratumor subgroup	Study number	Case number	HR (95%CI)-model	*P* value	Heterogenety	
					*I*^2^ (%)	*P*
CD8^+^						
Ethnicity						
Asian	9	1348	0.72 (0.60-0.87) - fixed	0.001	0	0.619
Caucasion	3	175	0.77 (0.46-1.29) - fixed	0.318	25.9	0.259
Sample size						
≥ 100	7	1183	0.74 (0.48-1.12) - fixed	0.001	0	0.445
< 100	5	340	0.73 (0.60-0.88) - fixed	0.155	0	0.528
Cut-off value						
Median	8	1133	0.73 (0.60-0.89) - fixed	0.002	0	0.504
Others	4	390	0.74 (0.50-1.08) - fixed	0.114	0	0.444
FOXP3^+^						
Ethnicity						
Asian	11	1265	1.66 (1.08-2.56) - random	0.022	73.0	<0.001
Caucasion	2	104	0.70 (0.33-1.48) – fixed	0.350	0	0.709
Sample size						
≥ 100	7	970	1.60 (1.09-2.36) - random	0.016	80.8	<0.001
< 100	6	399	1.23 (0.47-3.19) - random	0.617	58.5	0.025
Cut-off value						
median	9	991	1.66 (0.96-2.88) -random	0.072	78.0	<0.001
others	4	378	1.37 (0.93-2.03) – fixed	0.115	33.0	0.214

OS: overall survival; HR: hazard ratio; CI: confidence interval.

Four articles evaluated the OS of patients with high density of CD8^+^ TILs in ET [6, 21, 35], and the pooled analysis demonstrated that high density of infiltration of CD8^+^ T lymphocytes was statistically significant associated with better OS (pooled HR = 0.71; 95% CI = 0.53-0.97; *P* = 0.029) (Figure 2B).

### DFS

Five articles evaluated the DFS of patients with density of CD8^+^ T lymphocytes in IT [12, 17, 19, 23, 27]. The pooled analysis demonstrated that high density of CD8^+^ TILs in IT was statistically significant associated with better DFS (pooled HR = 0.57; 95% CI = 0.35-0.94; *P* = 0.029) (Figure 2B). However, CD8^+^ TILs in ET was not significantly associated with better DFS (*P* = 0.950)

### FOXP3^+^ T lymphocyte subset

A total of 14 articles, including 1,486 patients with gastric cancer, researched the clinical outcome of patients with high density of FOXP3^+^ TILs [6–8, 12, 14, 16, 17, 21, 23, 25, 31–33, 35]. Stratified by the endpoints, there were 12 of OS and 4 of DFS, respectively.

### OS

Twelve articles focused on the relationship between the density of FOXP3^+^ Treg in IT and OS [6–8, 14, 16, 17, 21, 25, 31–33, 35]. A random model was used because of a significant heterogeneity (*P* < 0.001, *I*^2^ = 71.6%), and the result demonstrated patients with high infiltration of FOXP3^+^ Treg had worse OS (pooled HR = 1.57; 95% CI = 1.04–2.37; *P* = 0.033) (Figure 2C). Subgroup analysis showed that patients with high density of FOXP3^+^ TILs had worse OS in the groups with Asian patients (pooled HR = 1.66, 95% CI = 1.08-2.56; *P* = 0.022) and large sample size (≥ 100; pooled HR = 1.60, 95% CI = 1.09-2.36; *P* = 0.016) (Table 3). We further performed a sensitivity analysis which demonstrated the result pattern was not impacted by any single study. The results of meta-regression showed that ethnicity (*P* = 0.860), sample size (*P* = 0.737) and cut-off value (*P* = 0.759) all had no statistically significant influence on the combined effect size for OS.

Four articles evaluated the OS of patients with high density of FOXP3^+^ TILs in ET [6, 14, 21], and the pooled analysis revealed that high density of infiltration of FOXP3^+^ TILs was associated with better OS (pooled HR = 0.76; 95% CI = 0.60-0.96; *P* = 0.022) (Figure 2C).

### DFS

Four articles researched the DFS of patients with high density of FOXP3^+^ TILs [12, 16, 17, 23]. Subgroup analysis demonstrated that FOXP3^+^ TILs was not related to DFS in IT or ET (*P* = 0.756, *P* = 0.500, respectively) (Figure 2C).

### CD3^+^ T lymphocyte subset

Seven articles researched clinical outcome of patients with high density of CD3^+^ TILs [5, 6, 11, 16, 17, 21, 29]. Subgroup analysis demonstrated that patients with high CD3^+^ T cells infiltration in IT had better OS (pooled HR = 0.52; 95% CI = 0.43-0.63; *P* < 0.001) (Figure 2D). In other subgroups, there was no statistical significance (Table 2).

### CD4^+^ T lymphocyte subset

Four articles researched clinical outcome of patients with high CD4^+^ T cells infiltration [17, 20, 21, 35]. However, subgroup analysis demonstrated that CD4^+^ T cells infiltration in IT/ET was not significantly associated with survival outcomes (Table 2).

### CD57^+^ NK cell subset

Four articles researched the clinical outcome of patients with high CD57^+^ NK cells infiltration [21, 29, 30, 34]. We found that CD57^+^ NK cells infiltration in IT was associated with better OS (pooled HR = 0.89; 95% CI = 0.85-0.94; *P* < 0.001). However, CD57^+^ NK cells infiltration in ET was not significantly associated with the survival outcome (Table 2).

### Other subsets

There were 3, 2, 2 and 2 articles investigated CD20^+^, CD45RO^+^, Granzyme B^+^ and T-bet^+^ TILs in patients with gastric cancer, respectively [5, 6, 12, 17, 19, 22, 24]. Subgroup analysis suggested that patients with high density of CD20^+^ TILs in IT had better DFS (pooled HR = 0.54; 95% CI = 0.34-0.86; *P* = 0.010); patients with high density of CD45RO^+^ TILs in IT had better OS (pooled HR = 0.56; 95% CI = 0.37-0.84; *P* = 0.005), and patients with high density of Granzyme B^+^ TILs in ET had better OS (HR = 0.35; 95% CI = 0.12-0.99; *P* = 0.050). Moreover, patients with high density of T-bet^+^ TILs in IT had better OS (HR = 0.55; 95% CI = 0.30-0.99; *P* = 0.047) and DFS (pooled HR = 0.54; 95% CI = 0.34-0.87; *P* = 0.012) (Table 2).

### Publication bias

**Figure 3:**
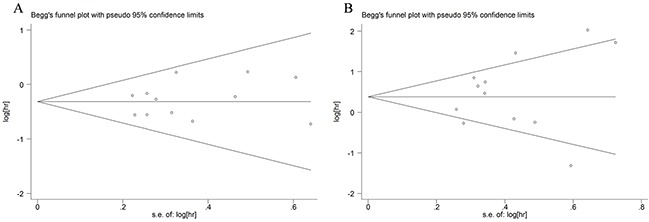
Funnel plots of CD8^+^ T cells (A) and FOXP3^+^ Tregs (B) infiltration in tumor nest

We only evaluated the publication bias of subgroups that contained over 10 studies. First, we use the funnel plot to detect the publication bias. The funnel plots of the CD8^+^ and FOXP3^+^ TILs in IT were substantially symmetric (Figure 3). Then, we used Begg's and Egger's tests to evaluated the publication bias. The *P* values were all greater than 0.05. Therefore, our meta-analysis has no publication bias.

## DISCUSSION

The prognostic value of TILs in gastric cancer was quantitatively evaluated in this meta-analysis. Considering the bidirectional role of TILs in tumor-associated immune responses, it is necessary to assess the prognostic role of different TIL subsets. Therefore, we conduct subgroup analyses based on subset of TILs.

Each subset of TILs plays its own roles in the development of gastric cancer, which contribute to their prognostic roles in gastric cancer. CD8, FOXP3, CD3, CD4, CD45RO, Granzyme B and T-bet are mainly located in the surface of T lymphocytes. Some of them are associated with favorable OS and/or DFS. CD8 and Granzyme B are surface antigens of cytotoxic T lymphocytes (CTLs) that are the main effective cells in the anti-tumor immune response [21]. This is in agreement with the results of our study in which these two subsets were associated with better OS and/or DFS. CD3 is a common surface antigen of T cells, and Arigami et al. reported the density of CD3^+^ TILs decreased during tumor progression [36]. It is noteworthy that the prognostic effect of CD3^+^ TILs in IT appeared most significant in our study. CD45RO is the most specific surface antigen of memory T cells, and this subset could prevent peritoneal dissemination of gastric cancer by inhibiting tumor growth and invasion [37]. T-bet is the most specific marker for T helper type 1 (Th1) cells, but besides Th1 cells, CD8^+^ CD4^+^ and CD56^+^ lymphocytes also express T-bet [24]. And for CD8^+^ CD4^+^ and CD56^+^ lymphocytes, T-bet is a T-box transcription factor which is crucial for the their differentiation and functions [38, 39].

Furthermore, other surface antigens located in T lymphocytes showed different prognostic roles. CD4^+^ lymphocytes include a group of heterogeneous T lymphocytes, which can secret diverse cytokines, and IL-17 is one of the most representative cytokines. Studies showed that IL-17 could promote tumor angiogenesis, proliferation, migration and invasion [40, 41]. On the contrary, many studies also confirmed IL-17 has anti-tumor effects. Bencherit et al. reported IL-17 could increase the infiltration and cytotoxicity of CTLs [42]. Antonysamy et al. found IL-17 could also increase the rate of mature and function of dendritic cells (DCs) [43]. Therefore, the roles of CD4^+^ TILs are very complicated [44], and in our study, the prognostic value of CD4^+^ TILs shows no significance. FOXP3 is the most specific marker on regulatory T cells (Tregs) that are generally considered to be immunosuppressive and block of effective antitumor immunity, therefore are associated with poor outcome in several kinds of tumors [45, 46]. But in our study, FOXP3^+^ TILs played bidirectional prognostic roles including positive effect in IT and negative effect in ET. That may be because FOXP3^+^ TILs in different anatomical locations may have opposite functions and influences between each other [21].

B cells and NK cells also play important roles in tumor-associated immune responses. CD20 generally exists in the surface of B cells. CD20^+^ B cells not only contribute to cellular immunity by providing costimulatory signals to T cells and/or serving as antigen-presenting cells, but also to humoral immunity by producing antibody [47, 48]. CD57 is a marker of NK cells which could attack tumor cells directly, and the recruitment of NK cells could exhibit strong antitumor activity and generate a better prognosis in gastric cancer [49, 50]. The functions of these two subsets were consistent with our results.

In addition to prognostic biomarker for gastric cancer, we observed that TILs are potential targets for immunotherapy. Immunotherapy has long been regarded as a powerful anti-tumor treatment which is less toxic and tumor-specific [51]. This treatment contains adoptive cell therapy, monoclonal antibody-based treatment and cancer vaccines. CD8^+^ TILs have crucial targets in these courses. By blocking the interaction of CD8^+^ T cell-related receptors (CTLA-4 and PD-1) and ligands (PD-L2 and PD-L1), anti-tumor immunity is enhanced in patients with advanced solid tumor, including gastric cancer [52]. Champiat et al. also confirmed that augmentation of the local immune response might be a promising target for new immunotherapy [53]. According to our study, the accumulation of CD8^+^, CD3^+^, CD57^+^ TILs and depletion of FOXP3^+^ TILs were all favorable prognostic factors in gastric cancer. Based on these studies, we propounded CD3^+^, CD57^+^ and FOXP3^+^ TILs could also serve as targets in immunotherapy like CD8^+^ TILs. However, we still need more prospective studies to verify the feasibility of other subsets in future gastric cancer immunotherapy.

To our knowledge, this is the first meta-analysis of evaluating the prognostic role of TILs in gastric cancer. The results from our study should be interpreted with caution for following reasons. First, some studies used different cut-off values that could reduce the practicability of TILs in the process of estimating prognosis. Second, when several HRs were not directly provided in the original studies, we obtained them by calculating the data extracted from the survival curves. Thus, small statistical errors were inevitable. Third, there are still some studies that only used univariate analysis, although univariate analysis may overestimate effect sizes compared to multivariate analysis under common settings. Finally, there are few investigations of some subsets such as CD45RO^+^, Granzyme B^+^ and T-bet^+^ TILs. This could result in relatively insufficient data in the subgroup analysis.

In conclusion, our meta-analysis provides strong evidence that TILs might serve as a robust marker for predicting the outcome of patients with gastric cancer. Especially, high density of FOXP3^+^ TILs in the ET was a negative prognostic factor, which was opposite to previous results [6, 54]. We still need more randomized controlled trials to further confirm the results of this meta-analysis.

## MATERIALS AND METHODS

We conducted this meta-analysis according to the Observational Studies in Epidemiology (MOOSE) guidelines [56] and the Systematic Reviews and Meta-Analyses (PRISMA) guidelines [55].

### Search strategy

We searched the literatures published prior to November 2016 through PubMed, Embase and the Cochrane. Keywords used in the search process were “TILs OR tumor-infiltrating lymphocyte OR intratumoral lymphocyte OR tumor infiltrating lymphocyte OR intra-tumoral lymphocyte OR intratumoral lymphocyte” (all fields) AND “stomach OR gastric” (all fields) AND “tumour OR tumor OR cancer OR neoplasm OR carcinoma” (all fields) AND “prognostic OR prognosis OR outcome OR survival” (all fields). We browsed not only the title and abstract but also the full text of identified articles. The availability evaluation and database search were conducted independently by two reviewers. The third reviewer resolved the disagreement from two reviewers.

### Inclusion and exclusion criteria

Only the literatures meeting the following criteria were eligible for inclusion: (1) studies researching the prognostic effect of TILs or associated TIL subsets in gastric cancer; (2) the expression of TILs or associated TIL subsets in IT or ET of patients with gastric cancer; (3) HR and 95% confidence intervals (CI) could be extracted; (4) the sample size of studies was greater than 50. Several literatures were excluded according to the following criteria: (1) studies with small sample size (< 50) were excluded to avoid the publication bias from too small sample size; (2) reviews, letters, case reports, animal trials and conference abstracts were excluded; (3) only the complete or most recent study was enrolled if one patient cohort were researched by multiple studies.

### Data extraction

The author information, publication year, TIL subsets and distribution site, origin of population, tumor stage, cut-off values, sample size, follow-up, detected methods, HRs and 95% CIs were collected by two researchers. OS and DFS (where possible) were chosen as the indexes of this meta-analysis. Recurrence-free survival (RFS) was used as a substitute if DFS was lacking because RFS is similar to DFS. Compared with univariate analysis, the result from multivariate analysis was preferred because it had improved precision after accounting for confounding factors.

### Statistical analysis

The prognostic effect of different levels of TILs in gastric cancer was evaluated by HRs and 95% CIs. If the HRs and 95% CIs were reported in the study, we extracted them directly. Otherwise, we obtained them by calculating the data extracted from Kaplan–Meier survival curves using Engauge Digitizer version 4.1 [57, 58]. The *I*^2^ statistic and Chi-square test (*P* value) were used to assess the statistical heterogeneity [59, 60]. Heterogeneity was suggested when *I*^2^ was below 50% and/or *P* value was greater than 0.05. The source of heterogeneity was explored primarily through meta-regression and subgroup analysis. Funnel plot, Egger's and Begg's tests were performed to evaluate the publication bias [61]. All analyses were performed using STATA version 12.0 (Stata Corporation, College Station, TX, USA).
